# Fungi Benefit from Two Decades of Increased Nutrient Availability in Tundra Heath Soil

**DOI:** 10.1371/journal.pone.0056532

**Published:** 2013-02-20

**Authors:** Riikka Rinnan, Anders Michelsen, Erland Bååth

**Affiliations:** 1 Department of Biology, Lund University, Lund, Sweden; 2 Department of Biology, University of Copenhagen, Copenhagen, Denmark; 3 Center for Permafrost (CENPERM), University of Copenhagen, Copenhagen, Denmark; University of California Irvine, United States of America

## Abstract

If microbial degradation of carbon substrates in arctic soil is stimulated by climatic warming, this would be a significant positive feedback on global change. With data from a climate change experiment in Northern Sweden we show that warming and enhanced soil nutrient availability, which is a predicted long-term consequence of climatic warming and mimicked by fertilization, both increase soil microbial biomass. However, while fertilization increased the relative abundance of fungi, warming caused only a minimal shift in the microbial community composition based on the phospholipid fatty acid (PLFA) and neutral lipid fatty acid (NLFA) profiles. The function of the microbial community was also differently affected, as indicated by stable isotope probing of PLFA and NLFA. We demonstrate that two decades of fertilization have favored fungi relative to bacteria, and increased the turnover of complex organic compounds such as vanillin, while warming has had no such effects. Furthermore, the NLFA-to-PLFA ratio for ^13^C-incorporation from acetate increased in warmed plots but not in fertilized ones. Thus, fertilization cannot be used as a proxy for effects on warming in arctic tundra soils. Furthermore, the different functional responses suggest that the biomass increase found in both fertilized and warmed plots was mediated via different mechanisms.

## Introduction

Microbial use of carbon substrates in soil is of critical importance when estimating impacts of climate change on the fate of soil carbon. Especially in the Arctic, where the amount of soil carbon exceeds the amount of carbon in aboveground plant biomass by an order of magnitude [1], microbial processes transforming the carbon compounds are crucial. In this region, climate change is predicted to increase the mean annual temperature by 2–9°C within this century [2].

Warming generally stimulates microbial growth [3] and thereby increases nutrient mineralization [4] in soil. As enhanced nutrient availability thus is an expected long-term consequence of warming, fertilization is often used as a long-term proxy for warming in experimental studies [5], [6]. Both warming and enhanced nutrient availability affect turnover of soil carbon, directly through effects on microbial activity [3] and indirectly by increasing plant growth [7] and altering vegetation composition [8], [9].

Changes in plant growth and vegetation composition influence carbon inputs to soil in several ways. In the short-term, the quantity and the chemical quality of root exudates may change as these differ among plant species [10]. Also, the distribution of these fresh carbon inputs in the soil horizon may change, as different plant groups have different rooting depths [6], [11]. In the long-term, vegetation changes will influence litter input. For example, an increase in deciduous shrubs [12] would lead to higher leaf litter deposition each autumn, while an increase in graminoids [8] would add easily decomposable plant litter to soil [13]. Climate change may also alter the chemical composition of a plant, often increasing the concentration of secondary metabolites in the plant tissue [14].

We aimed to unravel how the composition of microbial communities and their use of carbon substrates in subarctic tundra soil respond to long-term warming and enhanced nutrient availability using SIP-PLFA (stable isotope probing of phospholipid fatty acids). Soil samples were taken from a field experiment which had been running for 18 years prior to the sampling on a mesic/dry heath just above the tree line near Abisko Scientific Research Station in Northern Sweden. The warming treatment, which was accomplished by open-top chambers, initially resulted in an increased biomass of deciduous shrubs [15], decreased abundance of bryophytes [8], and reduced soil microbial biomass [5]. After a decade, warming had thus reduced the frequency of mosses by 50% and that of lichens by 35%, but there was no effect on graminoids [8]. The NPK (nitrogen, phosphorous and potassium) fertilization treatment, which aimed at mimicking increased soil nutrient availability, had also increased vascular plant biomass at the expense of bryophyte biomass [15], but increased soil microbial biomass including fungal biomass [5]. After 14 years, both warming and fertilization had increased the biomass of deciduous shrubs, but the increase in graminoids was still confined to the fertilized plots [15]. After 22 years, both warming and fertilization treatments had 35% higher vascular plant cover than the control [16].

In the laboratory, the soil samples were amended with a range of ^13^C-labelled substrates, and the uptake of these substrates into various fatty acids indicating different microbial groups and either growth (PLFAs) or storage (neutral lipid fatty acids, NLFAs) was followed. The selected substrates represented carbon sources present in soil. Glucose, acetic acid and glycine are simple compounds common in plant root exudates, and glycine is also a source of nitrogen for subarctic plants and microbes [11]. Starch is a very common polysaccharide in plant residues. Vanillin is a common product of lignin depolymerisation [17], containing a phenol ring, and is often used as a model substance indicating lignin degradation. Starch and vanillin are therefore examples of more complex substrates and supposedly more difficult to decompose.

We expected that, in line with the previous results from the field experiment [5], fertilization would increase and warming decrease microbial biomass. We hypothesized that fertilization would increase the uptake of carbon substrates into PLFA as compared to NLFA due to alleviated nutrient limitation. Furthermore, we expected that vanillin uptake would decrease with increased N availability, since it has earlier been shown that fertilization can suppress ligninolytic enzymes in forest soil [18]–[20]. Warming was expected to have the same effects as fertilization if this treatment would lead to increased nutrient availability.

## Results

### Treatment Effects on Fatty Acid Concentrations

**Figure 1:**
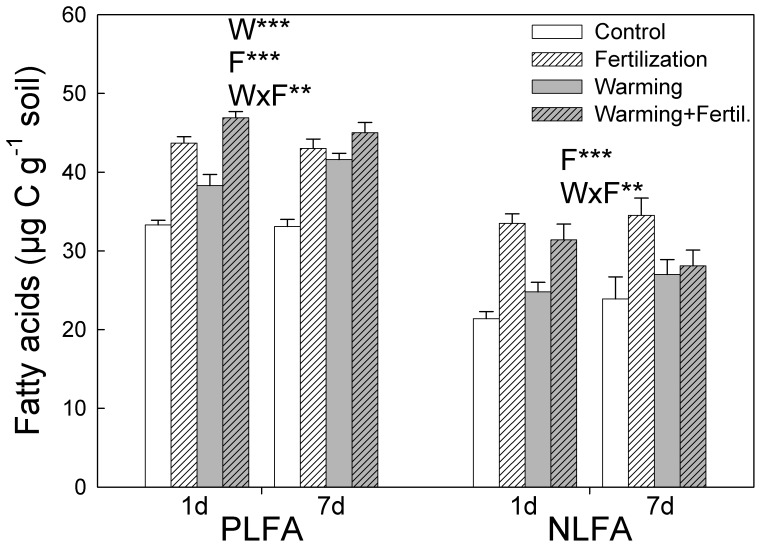
Total concentrations of phospholipid (PLFA) and neutral lipid fatty acids (NLFA) in tundra heath soil after 18 years of fertilization and warming treatments. The bars represent mean ± SE (n = 6) for 1-day and 7-day-long incubations averaged across the substrate additions, which did not affect the fatty acid concentrations. Significant effects of factors warming (W) and fertilizer addition (F), and their interaction are shown at **P<0.01, ***P<0.001 (Linear Mixed Models with Warming, Fertilization and Time as fixed factors and Block as a random factor).

Substrate additions did not cause any significant changes in the total PLFA or NLFA concentration, and therefore an average of each substrate is presented (Fig. 1). Fertilization and warming significantly increased the total PLFA concentration in soil by about 31% and 20%, respectively (Fig. 1; P<0.001), which indicates that these treatments increased microbial biomass. The total NLFA concentration was only increased by fertilization (P<0.001), although less when fertilization was combined with warming (Fig. 1; P<0.01 for warming×fertilization interaction). There were no significant differences between the two incubation times.

**Figure 2:**
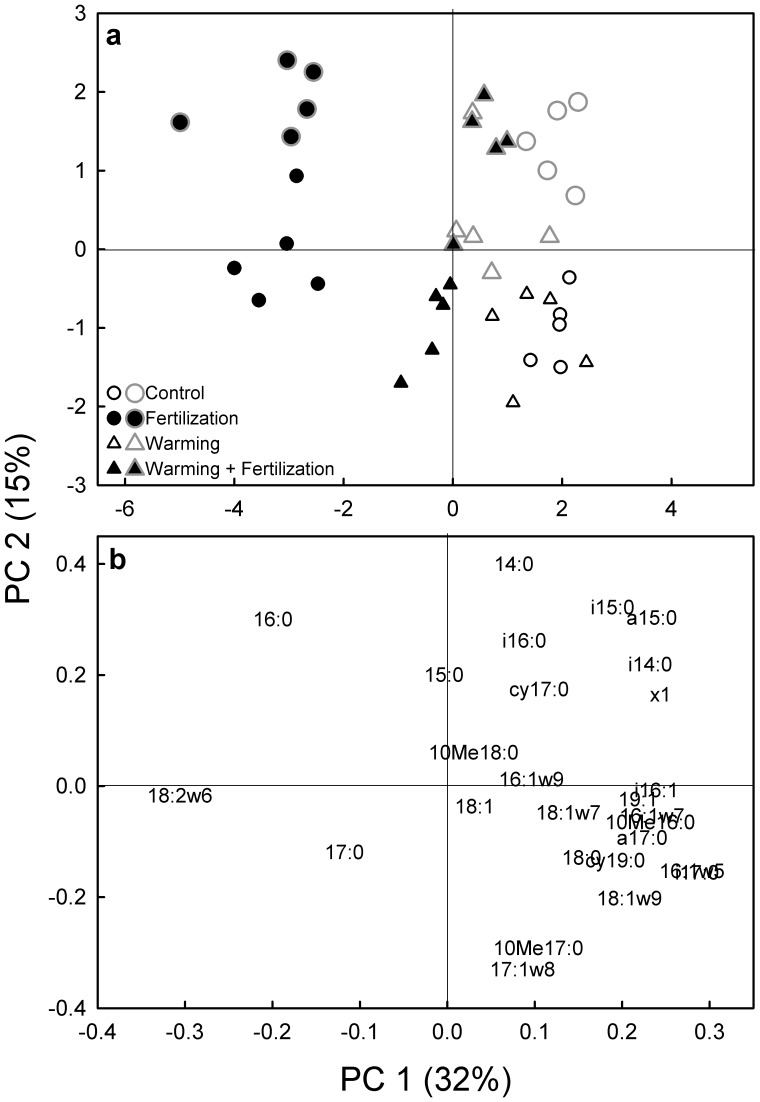
Principal component analysis of the phospholipid fatty acid (PLFA) mole percentage profiles in tundra heath soil from a field experiment with long-term fertilization and warming treatments. (a) A score plot showing mean values for substrate additions after a 1-day-long incubation (small symbols drawn with black line) and after a 7-day-long incubation (large symbols drawn with grey line). The mean values for all substrates within each treatment (glucose, glycine, acetic acid, starch and vanillin) are marked with similar symbols since the substrate additions did not affect the PLFA pattern. P<0.001 for effects of Warming, Fertilization, Time and Warming×Fertilization on the PC scores (Linear Mixed Models with Warming, Fertilization and Time as fixed factors and Block as a random factor). (b) A loading plot showing the individual PLFAs. Variance explained by principal component (PC) 1 and 2 in parentheses.

In the PCA of the PLFA profiles, the first PC (explained variance 32%) described treatment effects on the PLFA patterns (Fig. 2). The control soil had the highest values along this PC axis, followed by partly overlapping warming and warming + fertilization treatments close to the origin, and the fertilized soil had the lowest values (Fig. 2a; P<0.001 for effects of both warming and fertilization). The warming×fertilization interaction term was highly significant (P<0.001) because of the relatively similar PLFA patterns in the warming and warming + fertilization treatments as compared to fertilization alone. The fertilized soil was characterized by relatively higher amounts of the fungal biomarker 18∶2ω6,9 than the unfertilized soil (Fig. 2b). The second PC (explained variance 15%) mainly accounted for the difference between the incubation times (Fig. 2a; P<0.001); the relative abundance of the straight-chained PLFAs 14∶0, 15∶0 and 16∶0, and the markers for Gram-positive bacteria i14∶0, i15∶0, a15∶0 and i16∶0 increased from one to seven-day samples (Fig. 2b). The fungal biomarker 18∶2ω6,9 was not affected by the incubation time.

**Figure 3:**
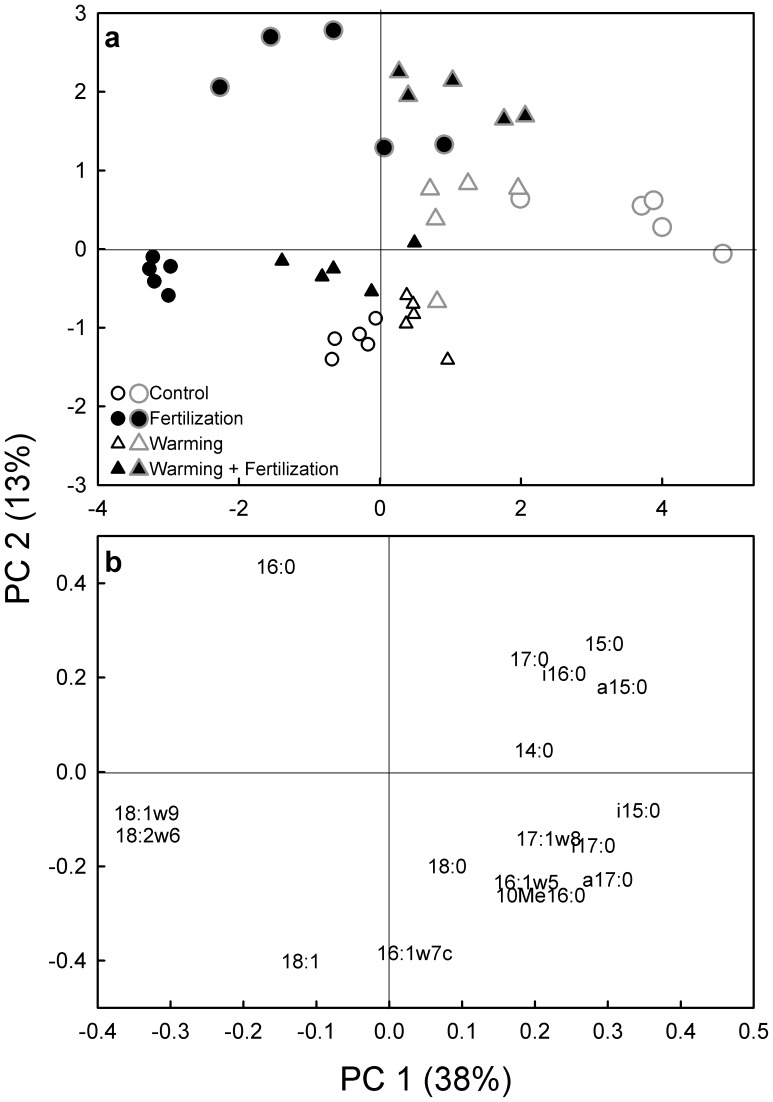
Principal component analysis of the NLFA mole percentage profiles in tundra heath soil from a field experiment with long-term fertilization and warming treatments. (a) A score plot showing mean values for substrate additions after a 1-day-long incubation (small symbols drawn with black line) and after a 7-day-long incubation (large symbols drawn with grey line). The mean values for all substrates within each treatment (glucose, glycine, acetic acid, starch and vanillin) are marked with similar symbols since the substrate additions did not affect the NLFA pattern. (b) A loading plot showing the individual NLFAs. Variance explained by principal component (PC) 1 and 2 in parentheses. P<0.001 for effects of Fertilization, Time and Warming×Fertilization on the PC scores (Linear Mixed Models with Warming, Fertilization and Time as fixed factors and Block as a random factor).

The PCA of the NLFA mole percentage profiles showed that while the 1-day incubation time produced relatively similar patterns except for the fertilization treatment, there was a large spread in the patterns for the 7-day-long incubation (Fig. 3a). As for PLFA, the first PC (explained variance 38%) accounted for the difference between the fertilized and the unfertilized soils, and showed a warming×fertilization interaction (Fig. 3a; P<0.001). As for PLFAs, the difference was mainly due to a higher abundance of the fungal markers, in this case the NLFAs 18∶2ω6,9 and 18∶1ω9 (Fig. 3b). The second PC mainly separated the incubation times from each other (Fig. 3a; P<0.001).

### Treatment Effects on ^13^C-incorporation into PLFA

There were no significant effects of treatments or incubation time on total ^13^C-incorporation from glucose, acetic acid, glycine (Table 1). Fertilization increased total ^13^C-incorporation from vanillin (P<0.001), but did not significantly affect incorporation from starch. The uptake of starch and vanillin increased from one to seven-day-long incubation (Table 1; P<0.01).

**Table 1:** Total ^13^C-incorporation into phospholipid (PLFA) and neutral lipid fatty acids (NLFA) and NLFA/PLFA ratios for ^13^C-incorporation into fungal biomarkers (mean±SE, *n* = 6) in tundra heath soil following incubation with added glucose, glycine, acetic acid, starch or vanillin for 1 or 7 days.

	1-day incubation				7-day incubation				Statistical
	C^a^	F	W	W+F	C	F	W	W+F	significance
Total ^13^C-incorporation into PLFA									
(µg ^13^C per mg added ^13^C g^−1^ soil)									
Glucose	1.48±0.13	1.35±0.06	1.19±0.12	1.27±0.12	1.45±0.20	1.23±0.06	1.32±0.08	1.40±0.14	n.s.^b^
Glycine	0.92±0.11	0.85±0.06	0.85±0.12	1.05±0.09	1.08±0.08	0.85±0.07	1.03±0.06	1.15±0.12	n.s.
Acetic acid	2.38±0.24	2.50±0.11	2.20±0.08	2.62±0.13	2.32±0.47	2.06±0.17	2.63±0.14	2.11±0.71	n.s.
Starch	0.40±0.06	0.33±0.06	0.28±0.03	0.39±0.08	1.24±0.04	1.20±0.11	1.03±0.07	1.36±0.25	T***
Vanillin	0.32±0.03	0.41±0.01	0.24±0.01	0.73±0.16	0.54±0.08	0.89±0.09	0.48±0.05	0.57±0.11	F***, T**
Total ^13^C-incorporation into NLFA									
(µg ^13^C per mg added ^13^C g^−1^ soil)									
Glucose	0.51±0.06	0.70±0.08	0.51±0.04	0.43±0.07	0.26±0.06	0.31±0.05	0.32±0.07	0.24±0.02	T***
Glycine	0.15±0.02	0.14±0.02	0.23±0.05	0.21±0.03	0.08±0.02	0.07±0.01	0.11±0.03	0.13±0.05	T**
Acetic acid	1.27±0.11	1.45±0.13	1.51±0.12	1.41±0.18	0.51±0.20	0.52±0.12	0.99±0.11	0.65±0.19	T***
Starch	0.06±0.01	0.11±0.02	0.08±0.01	0.12±0.01	0.12±0.05	0.10±0.01	0.15±0.03	0.15±0.01	n.s.
Vanillin	0.05±0.01	0.12±0.01	0.10±0.04	0.10±0.02	0.07±0.04	0.19±0.02	0.05±0.01	0.08±0.02	F**
NLFA/PLFA for ^13^C-incorporation from									
Glucose to 18∶2ω6,9	0.09±0.01	0.15±0.03	0.15±0.03	0.11±0.03	0.08±0.02	0.11±0.02	0.12±0.04	0.10±0.02	n.s.
Glucose to 18∶1ω9	1.09±0.16	1.78±0.15	1.65±0.25	1.25±0.09	0.58±0.07	0.57±0.06	0.80±0.15	0.68±0.02	T***
Acetic acid to 18∶2ω6,9	0.16±0.01	0.12±0.02	0.15±0.02	0.12±0.02	0.05±0.02	0.08±0.01	0.15±0.03	0.15±0.01	W**, W×T**
Acetic acid to 18∶1ω9	1.65±0.23	1.53±0.20	2.21±0.42	1.68±0.26	0.55±0.03	0.60±0.09	0.88±0.05	0.71±0.02	T***

a C, control; F, fertilization; W, warming; W+F, warming+fertilization.

b n.s., no significant effects at *P*<0.01.

Significant effects of factors warming (W), fertilizer addition (F), time (T) and their interactions are shown at **P<0.01, ***P<0.001.

**Figure 4:**
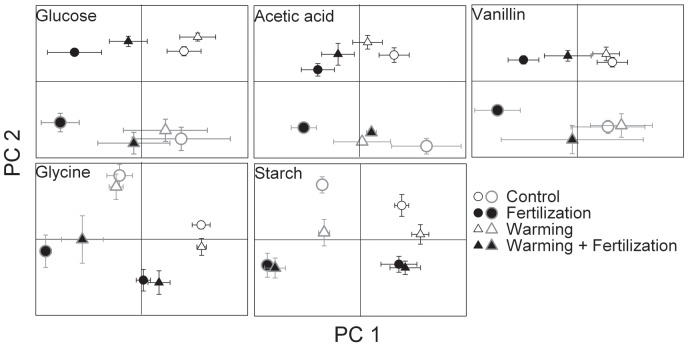
Principal component analysis (PCA) of the relative ^13^C-incorporation of added ^13^C-labelled glucose, glycine, acetic acid, starch and vanillin into phospholipid fatty acids (PLFAs) of tundra heath soil. Scores (mean ± SE) of the principal components (PC) 1 and 2 showing effects of warming and fertilization, and differences between the incubation times (1day, small symbols drawn with black line; 7 days, large symbols drawn with grey line). The PCAs were run separately for each substrate. The explained variances were 33–46% for PC1 and 18–27% for PC2. For glucose, glycine, acetic acid and vanillin P<0.001 for Fertilization and Time effect on the PC scores (Linear Mixed Models with Warming, Fertilization and Time as fixed factors and Block as a random factor). For starch P<0.01 for Warming, P<0.001 for Fertilization and Time, and P<0.01 for Warming×Fertilization.

The PCAs for the ^13^C-incorporation patterns showed that for all substrate additions, the first two PCs separated the fertilized soil from the unfertilized soil (Fig. 4; P<0.001). A common explanation for this difference was an increased incorporation of ^13^C into the fungal biomarker PLFAs, 18∶2ω6,9 and 18∶1ω9 and a decreased incorporation into most of the bacterial biomarker PLFAs under fertilization (see Figs S1, S2, S3, S4, S5, S6, S8). As an exception, the Gram-negative bacteria indicated by cy17∶0 and cy19∶0 took up significantly more glycine, starch and vanillin in the fertilized than in the unfertilized soil (Fig. S3). In addition, utilization of starch was higher in the fertilized than in the unfertilized soil for Gram-positive bacteria containing i16∶0 and the actinomycetes indicated by the methylated PLFA 10Me16∶0, especially after incubation for seven days (Figs S6 and S7).

The substrate utilization patterns in the warming treatment were rather similar to those in the control samples, except for starch addition with a significant warming effect on the second PC (Fig. 4, P<0.01). The incorporation of ^13^C from starch into the PLFAs i16∶0 and 10Me18∶0 was higher in the warming treatment than in the control soil after incubation for seven days (Figs S6 and S8). Uptake of glucose by organisms containing the PLFA 18∶1ω9 was significantly reduced by warming (Fig. S2). The warming+fertilization treatment had relatively similar substrate utilization patterns as fertilization alone (Fig. 4), which led to warming×fertilization interactions in the incorporation to some individual PLFAs (Figs S1, S2, S3, S4, S5, S6, S7, S8).

A clear difference in the ^13^C-incorporation patterns between the two incubation times could be observed on the first two PCs for all substrates (Fig. 4; P<0.001). This owed mainly to the increased incorporation into the PLFAs cy17∶0, cy19∶0, 10Me16∶0 and 10Me18∶0 over time (Figs S3, S7, S8).

### Treatment Effects on ^13^C-incorporation into NLFA

The incorporation of ^13^C to NLFA decreased over time for glucose, acetic acid, and glycine (Table 1; P<0.01). Fertilization increased the total incorporation of ^13^C from vanillin (P<0.01) but did not significantly affect incorporation from other substrates.

**Figure 5:**
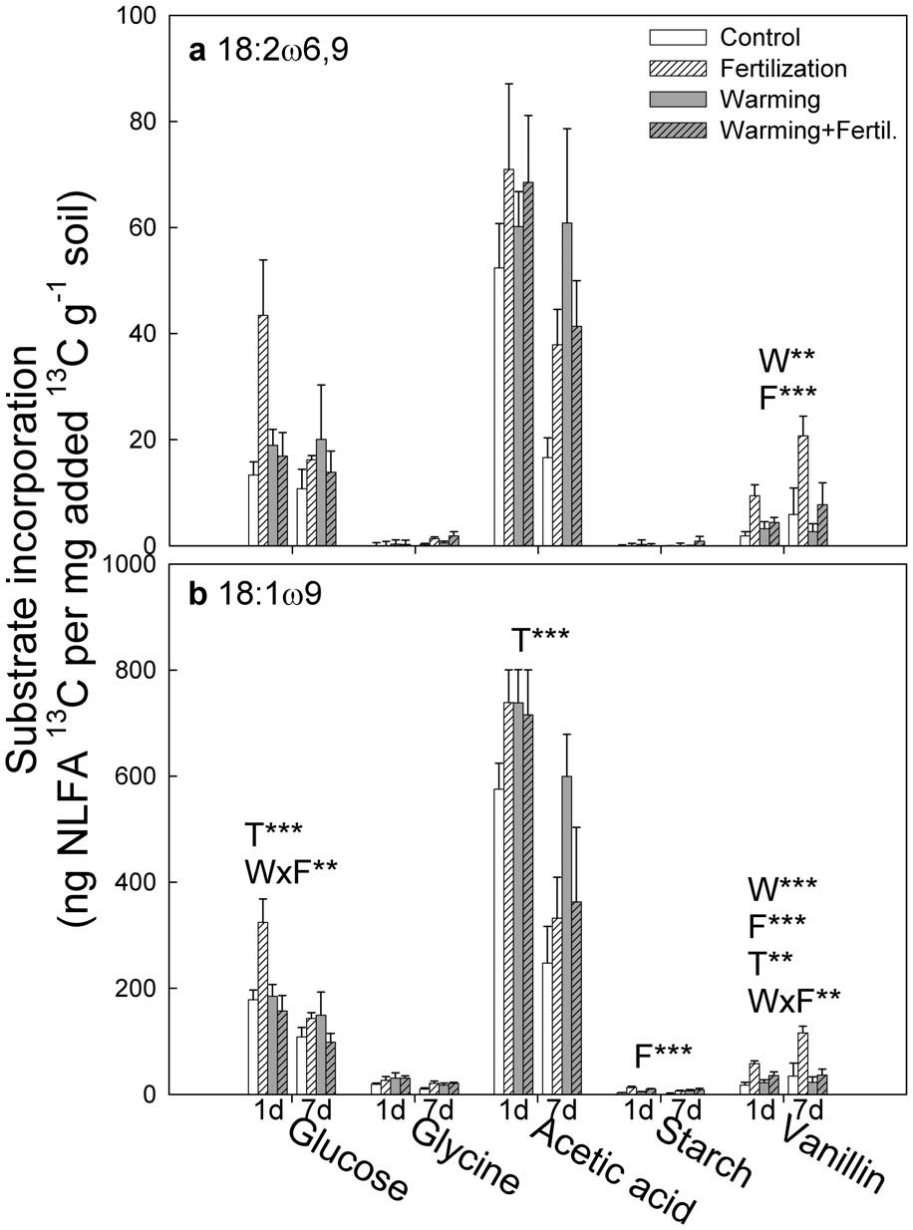
Incorporation of ^13^C from glucose, glycine, acetic acid, starch and vanillin into the individual NLFAs (a) 18∶2ω6,9 and (b) 18∶1ω9. Tundra heath soils from a field experiment with long-term warming and fertilization treatments were incubated 1 and 7 days.The bars represent mean+SE. Note different y-axis scales. Significant effects of factors warming (W), fertilizer addition (F), time (T) and their interactions for each substrate are shown at **P<0.01, ***P<0.001 (Linear Mixed Models with Warming, Fertilization and Time as fixed factors and Block as a random factor).

Incorporation of ^13^C to the fungal biomarker NLFAs, 18∶2ω6,9 and 18∶1ω9, was highest from acetic acid, followed by glucose and vanillin (Fig. 5a, b). Incorporation of ^13^C to these NLFAs from glycine and starch was minimal. The response patterns for 18∶2ω6,9 and 18∶1ω9 were similar, but because of the higher variance, only vanillin-amended samples showed significant differences at P<0.01 for 18∶2ω6,9; fertilization increased (P<0.001) and warming decreased (P<0.01) ^13^C-incorporation from vanillin (Fig. 5a). Similar responses were observed for ^13^C-incorporation from vanillin into the NLFA 18∶1ω9 (Fig. 5b; P<0.001 for both warming and fertilization). Fertilization also significantly increased ^13^C-incorporation from glucose, when not combined with warming treatment (P<0.01 for warming×fertilization).

The NLFA-to-PLFA ratios for ^13^C-incorporation into 18∶2ω6,9 and 18∶1ω9 were not significantly affected by treatments for other substrates than acetic acid (data for glucose and acetic acid shown in Table 1). For acetic acid, the ratio for incorporation into 18∶2ω6,9 was significantly increased by warming after incubation for 7 days (with a similar insignificant trend for 18∶1ω9), while it was unaffected by fertilization (Table 1).

## Discussion

Fertilization of the subarctic heath for 18 years had increased the total PLFA concentration in soil by 31%, which indicates increased microbial biomass. This increase is in agreement with the results after 15 treatment years, when fertilization also had increased the total PLFA concentration, however only by 16% [5]. In contrast to the reduction of total PLFA by the warming treatment after 15 years [5], warming for the additional three years up to the sampling for the present study had led to a significantly increased total PLFA concentration. This implies that the total microbial biomass responses in the warming treatment now, for the first time, resemble those in the fertilization treatment [21]. The result probably reflects the long-term increase in the total cover of vascular vegetation. After 22 years, both warming and fertilization treatments had 35% higher vascular plant cover than the control [16], in contrast to previous measurements after 5 and 10 years, with varying responses in the two treatments [22]. Increased primary production typically leads to an increase in soil microbial biomass [23], thanks to enhanced substrate inputs.

Based on a PCA of the PLFA profiles, the microbial community composition in the fertilized soil was characterized by a significantly higher abundance of the fungal biomarker, 18∶2ω6,9, as compared to the unfertilized soil, which was similar to the results after 15 treatment years [5]. The fertilization-induced increase in fungi was further evidenced by the significantly higher total concentration of NLFAs, which are mainly found in eukaryotic organisms and thus are indicative of fungi in soil [24]. More specifically, the fungal marker NLFAs, 18∶2ω6,9 and 18∶1ω9 [24] had a significantly higher relative abundance in the fertilized than in the unfertilized soil. Rinnan et al. [5] discussed possible effects of adding N on soil fungi, also comparing with other habitats like coniferous forest soil. They suggested that the fertilizer-induced increase in fungi in this peaty subarctic soil resulted from 1) an alleviated N or P limitation of the fungal exoenzyme production, 2) a greater predation pressure on the bacterial than on the fungal community or 3) a higher abundance of mycorrhizal fungal mycelium. An altered community composition of ectomycorrhizal fungi was also found in a fertilization experiment in Arctic soils in North America [25].

Direct effects of a higher temperature on the PLFA pattern is well studied, with increased length and lower degree of unsaturation of fatty acids at higher temperatures [26]. Such changes were not found in the present study, indicating no direct effect of temperature. Instead warming caused only a minimal shift in the fatty acid profiles, and the effect of warming overrode that of fertilization as the combined warming+fertilization treatment had a similar PLFA profile to warming alone. Thus, although warming had increased the soil microbial biomass (total PLFA) indicating similar effects as fertilization, the different responses in the community composition suggest that the mechanisms behind the increase in microbial biomass under these treatments are different. This is in line with the vegetation responses, which show that despite the identical increase in the total cover of vascular plants in warming and fertilization treatments, the responses in the dominant plant species under these treatment were contrasting. While warming enhanced the cover of ectomycorrhizal *Betula nana*, fertilization benefited the ericoid mycorrhizal *Empetrum hermaphroditum* [16]. It is highly unlikely that the air warming by open-top chambers has had any direct effect on soil microbial communities in the long term, because the expansion of the vascular plant cover has over time led to shading and no soil temperature differences between the control and warming treatments [27].

The most obvious difference between the warming and fertilization treatments is that the supplied NPK fertilization provides the microbes with extra N and P, alleviating limitation by these nutrients, whereas soil warming increases mineralization of carbon as well as other nutrients. Earlier results have shown that microbes are N-limited or limited by both C and N in northern soils [6], [28], including soils from the Abisko region [3], [5]. Findings from another experiment in Abisko suggest, however, that warming shifts the originally N-limited soil microbial community slightly towards carbon limitation, although no significant changes in mineralization are observed [3]. This indicates that the warming-induced increase in net mineralization may be small, as also suggested by *in situ* buried bag studies at the experimental site [29], [30].

Utilization of the amended substrates by soil microbial communities was mainly affected by fertilization, while warming only had few effects. The most pronounced response was that the fertilized soil was characterized by a relatively higher ^13^C-incorporation into fungi than into bacteria as compared with the unfertilized soil, irrespective of the added substrate. Incorporation into the fungal NLFA 18∶2ω6,9 followed a similar response, but because of high variance, only vanillin showed a statistically significant fertilization effect. The ^13^C-recovery in the fatty acid 18∶1ω9, which also indicates fungi [24], was increased by fertilization for vanillin (both PLFA and NLFA) and starch (only NLFA).

Fungi store carbon in excess as triacylglycerols, which are detected in the NLFA fraction [24]. Allocation to fungal energy storage can thus be estimated with NLFA-to-PLFA ratios, and a lower ratio after adding C-rich, labile, substrates indicates available N in the soil [24]. Contrary to our hypothesis, there were no significant fertilization effects on the NLFA-to-PLFA ratios for ^13^C-incorporation. However, the NLFA-to-PLFA ratios for ^13^C-incorporation from acetic acid into 18∶2ω6,9 and 18∶1ω9 were higher in the warmed soil but unaffected by fertilization compared to the control. The increased allocation to storage under warming suggests that the increased temperature actually led to limitation of growth by the lack of nutrients when a simple carbon-rich substrate, acetic acid, was easily available. The contrasting responses of warming and fertilization clearly show that fertilization cannot be used as a proxy for effects of warming, as also indicated by the PLFA pattern.

That fertilizer addition increased ^13^C-incorporation from vanillin (a model substance indicating lignin depolymerisation), while warming had no significant effect, is additional evidence for this conclusion. This is also in agreement with the results of Neff et al. [31], who observed that fertilization of an alpine meadow had triggered increased decomposition of plant-derived lignin compounds. White-rot fungi of coniferous forest soil are known to down-regulate the production of lignolytic extracellular enzymes under high soil N concentrations [32], [33], suggesting that N fertilization should result in decreased lignin degradation. This was not the case in the present study. However, the increased vanillin utilization was mainly due to the stimulated uptake of ^13^C to the cyclic and fungal fatty acids, suggesting that both bacteria and fungi were involved in vanillin degradation in the soil studied.

The pattern of ^13^C incorporation from starch into PLFAs differed from the other substrates, which was also reported earlier [34]. Thus, although starch degradation is fairly common among microorganisms [35], a different subset of microorganisms appears to degrade starch as compared to the other studied substrates. Also, in contrast to the other substrates used in this study, utilization of starch was significantly affected by both fertilization and warming. More specifically, the incorporation of ^13^C from starch into indicators of Gram-positive bacteria (i16∶0) and actinomycetes (10Me16∶0 for fertilization and 10Me18∶0 for warming) increased by the treatments. It appears that the starch-degrading microorganisms were favored by the changes that had occurred both in the fertilized and warmed plots, possibly by the increase in deciduous shrubs and the concomitant increase in leaf litter [8], [15].

Increased biomass and activity of soil fungi relative to those of bacteria in response to enhanced nutrient availability may lead to altered ecosystem functioning. Most importantly, an increased importance of fungi relative to bacteria has been suggested to result in an increased carbon sequestration in soil ([36], but see also discussion by [37]). Because of the enormous size of the arctic soil carbon pool, even tiny changes in soil carbon processing have a potential to feed back on climate change. Effects of climate change on microbial use of carbon substrates would influence soil carbon storage both directly, as altered efflux of carbon from the soil, and indirectly, via priming effects (for a review, see [38]) that could lead to altered degradation of older carbon.

Several of our results indicate that – at least in the 18-year time horizon – warming does not result in similar effects as experimentally increased soil nutrient availability. Thus, climate warming will not increase carbon sequestration in tundra heaths via beneficial effects on soil fungi to the same degree as fertilization unless soil nutrient availability increases more than the increase in moderately warmer soil. As the N deposition rates in the region north of 60°N are low [39], a pronounced increase in nutrient availability via atmospheric deposition is, however, unlikely.

## Materials and Methods

### Experimental Design and Soil Sampling

A long-term climate change simulation experiment has been maintained on a tree-line heath at Abisko, northern Sweden (68°21′N, 18°49′E; 450 m a.s.l.), since 1989. The vegetation is dominated by ericoid dwarf shrubs (mainly *Cassiope tetragona* (L.) D. Don) and mosses, with deciduous shrubs and graminoids as subdominants. The experiment hosts various treatments (originally described in [21]) of which 1) warming, 2) fertilization, 3) the combination of warming and fertilization and 4) unmanipulated control plots were selected for the present study. For the warming treatment, 3–4°C air and 1–2°C soil temperature (4 cm depth; [40], [41]) increases were achieved with the help of dome-shaped open-top plastic greenhouses, that were erected on the plots for the growing season from early June to late August. The fertilization treatment simulated enhanced nutrient availability and it was maintained by annual additions of 10 g m^−2^ N, 2.6 g m^−2^ P and 9 g m^−2^ K as NH_4_NO_3_, KH_2_PO_4_ and KCl (except 1989, when half of this amount was applied, and 1993 and 1998, when no fertilization was performed). Each of the plots covered a 1.2×1.2 m area and was randomly distributed in six blocks, i.e. there were six replicate plots of each treatment. No specific permits were required for the described field studies, as the location is not privately-owned and not protected and the field studies did not involve endangered or protected species.

Three soil cores were taken from each field plot on August 24, 2006, i.e. after 18 years of treatments. The 5-cm-deep cores with a diameter of 4 cm were mixed into a single sample per treatment plot (total volume of 63 cm^3^), while removing roots and stones by hand sorting. The soil has a c. 15 cm deep organic layer with a pH of 7.1, which has not been affected by the treatments [41] and a soil organic matter content of 89% in the unfertilized and 92% in the fertilized soil in the top 5 cm [5]. The organic layer rests on rocky mineral soil without permafrost.

### Incubation with ^13^C-labelled Substrates

Uptake of label from ^13^C-labelled substrates into fatty acids was determined by SIP-PLFA as described by [34]. Five different substrates were used: universally ^13^C-labelled glucose (99%), glycine (99%) and starch (98%), ring-labelled vanillin (99%) and a 1∶1 mixture of acetic acid labelled with ^13^C in either one or the other of the two C atoms (Cambridge Isotope Laboratories, Andover, MA, USA). Subsamples of 0.4-g wet soil were distributed into ten small minigrip bags (duplicates of each substrate), and amended with 100 µl water containing a substrate. The added amount was 0.5 mg substrate g^−1^ wet soil (≈2 mg substrate g^−1^ d.w. organic matter), which corresponds to 0.21 mg ^13^C for glucose, 0.17 mg ^13^C for glycine, 0.11 mg ^13^C for acetic acid, 0.23 mg ^13^C for starch, and 0.25 mg ^13^C for vanillin per gram wet soil.

Half of the subsamples were incubated for 24 h and another half for 7 days, to enable comparison of immediate with longer-term uptake, at 15°C in dark followed by freezing and freeze-drying.

### Fatty Acid Analysis

Fatty acids were extracted from the freeze-dried soil following a modified Bligh and Dyer method according to [42] as described in [34]. Shortly, lipids were extracted and fractionated on silicic acid columns (Bond Elut, Varian Inc., Palo Alto, CA, USA) with chloroform (neutral lipids, the NLFA fraction), acetone (glycolipids) and methanol (phospholipids, the PLFA fraction). The NLFA and PLFA fractions were subjected to mild alkaline methanolysis, and the resulting fatty acid methyl esters were analyzed on a Hewlett-Packard 6890 gas chromatograph equipped with a 50-m HP5 capillary column (Hewlett-Packard, Palo Alto, CA, USA) and helium as the carrier gas. The GC was interfaced with a Europa 20/20 isotope ratio mass spectrometer (Sercon Ltd., Cheshire, UK) used for determination of the δ^13^C values [43]. Methyl nonadecanoate fatty acid (19∶0) was used as an internal standard.

Isotope data was calculated following Boschker [44]. The δ^13^C values of each lipid were corrected for the methyl group added during methanolysis [45]. For each PLFA and NLFA, the absolute amount of ^13^C incorporated was calculated by relating the increase in the fraction ^13^C after labeling [F = R/(R+1), where R is a ^13^C/^12^C-ratio] to the fatty acid concentration and the amount of ^13^C in the added substrate.

The fatty acids are presented as the total number of carbon atoms followed by a colon and the number of double bonds. The prefixes a and i signify anteiso- and isobranching, respectively. The prefix cy indicates cyclopropyl fatty acids, while 10Me is a methyl group on the 10th carbon atom from the carboxyl end of the molecule. Terminally and mid-chain branched fatty acids (i15∶0, a15∶0, i16∶0, 10Me16∶0, i17∶0, a17∶0, 10Me17∶0, 10Me18∶0), cyclopropyl saturated (cy-17∶0 and cy-19∶0) and some monounsaturated (16∶1ω7 and 18∶1ω7) fatty acids were considered indicative of bacteria [46]. The fatty acid 18∶2ω6,9 was considered to represent fungi [46]. The PLFA 18∶1ω9 is mainly of fungal origin (see discussion in [47]), and the NLFA 18∶1ω9 is of fungal origin, and often found to increase under excess of carbon [24].

### Statistical Analysis

The fatty acid concentrations and the total ^13^C-incorporation to fatty acids were analyzed for treatment effects by Linear Mixed Models of SPSS 14.0 for Windows with warming, fertilization, substrate and incubation time as fixed factors. The block variable from the field experiment was used as a random factor. As the differences between the ^13^C-labelled substrates were not of primary interest in the present study and have been described before [34], the analysis was run separately for each substrate. To reduce the chance for type I errors, the alpha-level of 0.01 was used to indicate statistical significance.

The unit-variance scaled and centered fatty acid mole percentage profiles and the patterns of ^13^C-incorporation were subjected to principal component analyses (PCA) using Simca-P 11.0 (Umetrics, Umeå, Sweden). The extracted principal components (PC) were analyzed for treatment effects using Linear Mixed Models in a similar manner as described above.

## Supporting Information

Figure S1 **Incorporation of ^13^C from glucose, glycine, acetic acid, starch and vanillin into the PLFA 18∶2ω6,9.** Tundra heath soils from a field experiment with long-term warming and fertilization treatments were incubated 1 and 7 days. The bars represent mean+SE. Note different y-axis scales. Significant effects of factors warming (W), fertilizer addition (F), time (T) and their interactions for each substrate are shown at **P<0.01, ***P<0.001 (Linear Mixed Models with Warming, Fertilization and Time as fixed factors and Block as a random factor).
(TIF)

Figure S2 **Incorporation of ^13^C from glucose, glycine, acetic acid, starch and vanillin into the PLFA 18∶1ω9.** Tundra heath soils from a field experiment with long-term warming and fertilization treatments were incubated 1 and 7 days. The bars represent mean+SE. Note different y-axis scales. Significant effects of factors warming (W), fertilizer addition (F), time (T) and their interactions for each substrate are shown at **P<0.01, ***P<0.001 (Linear Mixed Models with Warming, Fertilization and Time as fixed factors and Block as a random factor).
(TIF)

Figure S3 **Incorporation of ^13^C from glucose, glycine, acetic acid, starch and vanillin into the PLFA cy19∶0.** Tundra heath soils from a field experiment with long-term warming and fertilization treatments were incubated 1 and 7 days. The bars represent mean+SE. Note different y-axis scales. Significant effects of factors warming (W), fertilizer addition (F), time (T) and their interactions for each substrate are shown at **P<0.01, ***P<0.001 (Linear Mixed Models with Warming, Fertilization and Time as fixed factors and Block as a random factor).
(TIF)

Figure S4 **Incorporation of ^13^C from glucose, glycine, acetic acid, starch and vanillin into the PLFA 18∶1ω7.** Tundra heath soils from a field experiment with long-term warming and fertilization treatments were incubated 1 and 7 days. The bars represent mean+SE. Note different y-axis scales. Significant effects of factors warming (W), fertilizer addition (F), time (T) and their interactions for each substrate are shown at **P<0.01, ***P<0.001 (Linear Mixed Models with Warming, Fertilization and Time as fixed factors and Block as a random factor).
(TIF)

Figure S5 **Incorporation of ^13^C from glucose, glycine, acetic acid, starch and vanillin into the PLFA i15∶0.** Tundra heath soils from a field experiment with long-term warming and fertilization treatments were incubated 1 and 7 days. The bars represent mean+SE. Note different y-axis scales. Significant effects of factors warming (W), fertilizer addition (F), time (T) and their interactions for each substrate are shown at **P<0.01, ***P<0.001 (Linear Mixed Models with Warming, Fertilization and Time as fixed factors and Block as a random factor).
(TIF)

Figure S6 **Incorporation of ^13^C from glucose, glycine, acetic acid, starch and vanillin into the PLFA i16∶0.** Tundra heath soils from a field experiment with long-term warming and fertilization treatments were incubated 1 and 7 days. The bars represent mean+SE. Note different y-axis scales. Significant effects of factors warming (W), fertilizer addition (F), time (T) and their interactions for each substrate are shown at **P<0.01, ***P<0.001 (Linear Mixed Models with Warming, Fertilization and Time as fixed factors and Block as a random factor).
(TIF)

Figure S7 **Incorporation of ^13^C from glucose, glycine, acetic acid, starch and vanillin into the PLFA 10Me16∶0.** Tundra heath soils from a field experiment with long-term warming and fertilization treatments were incubated 1 and 7 days. The bars represent mean+SE. Note different y-axis scales. Significant effects of factors warming (W), fertilizer addition (F), time (T) and their interactions for each substrate are shown at **P<0.01, ***P<0.001 (Linear Mixed Models with Warming, Fertilization and Time as fixed factors and Block as a random factor).
(TIF)

Figure S8 **Incorporation of ^13^C from glucose, glycine, acetic acid, starch and vanillin into the PLFA 10Me18∶0.** Tundra heath soils from a field experiment with long-term warming and fertilization treatments were incubated 1 and 7 days. The bars represent mean+SE. Note different y-axis scales. Significant effects of factors warming (W), fertilizer addition (F), time (T) and their interactions for each substrate are shown at **P<0.01, ***P<0.001 (Linear Mixed Models with Warming, Fertilization and Time as fixed factors and Block as a random factor).
(TIF)

## References

[1] Jonasson S, Chapin FS,III, Shaver GR (2001) Biogeochemistry in the Arctic: Patterns, processes and controls. In: Schulze E-D, Heimann M, Harrison S, Holland E, Lloyd J, Prentice IC, Schimel D, editors. Global Biogeochemical Cycles in the Climate System. San Diego: Academic Press. 139–150.

[2] IPCC (2007) Climate change 2007: the physical science basis. Contribution of working group I to the fourth assessment report of the intergovernmental panel on climate change. Cambridge: Cambridge University Press.

[3] Rinnan R, Michelsen A, Bååth E, Jonasson S (2007) Mineralization and carbon turnover in subarctic heath soil as affected by warming and additional litter. Soil Biol Biochem 39: 3014–3023.

[4] Rustad LE, Campbell JL, Marion GM, Norby RJ, Mitchell MJ, et al (2001) A meta-analysis of the response of soil respiration, net nitrogen mineralization, and aboveground plant growth to experimental ecosystem warming. Oecologia 126: 543–562. 28547240 10.1007/s004420000544

[5] Rinnan R, Michelsen A, Bååth E, Jonasson S (2007) Fifteen years of climate change manipulations alter soil microbial communities in a subarctic heath ecosystem. Global Change Biol 13: 28–39.

[6] Mack MC, Schuur EAG, Bret-Harte MS, Shaver GR, Chapin FS (2004) Ecosystem carbon storage in arctic tundra reduced by long-term nutrient fertilization. Nature 431: 440–443. 15386009 10.1038/nature02887

[7] van Wijk MT, Clemmensen KE, Shaver GR, Williams M, Callaghan TV, et al (2004) Long-term ecosystem level experiments at Toolik Lake, Alaska, and at Abisko, Northern Sweden: generalizations and differences in ecosystem and plant type responses to global change. Global Change Biol 10: 105–123.

[8] Graglia E, Jonasson S, Michelsen A, Schmidt IK, Havström M, et al (2001) Effects of environmental perturbations on abundance of subarctic plants after three, seven and ten years of treatments. Ecography 24: 5–12.

[9] Walker MD, Wahren CH, Hollister RD, Henry GHR, Ahlquist LE, et al (2006) Plant community responses to experimental warming across the tundra biome. P Natl Acad Sci Biol 103: 1342–1346. 10.1073/pnas.0503198103 PMC1360515 16428292

[10] Bais HP, Weir TL, Perry LG, Gilroy S, Vivanco JM (2006) The role of root exudates in rhizosphere interactions with plants and other organisms. Annu Rev Plant Biol 57: 233–266. 16669762 10.1146/annurev.arplant.57.032905.105159

[11] Andresen L, Jonasson S, Ström L, Michelsen A (2008) Uptake of pulse injected nitrogen by soil microbes and mycorrhizal and non-mycorrhizal plants in a species-diverse subarctic heath ecosystem. Plant Soil 313: 283–295.

[12] Sturm M, Racine C, Tape K (2001) Climate change: Increasing shrub abundance in the Arctic. Nature 411: 546–547. 11385559 10.1038/35079180

[13] Cornelissen JH, Van Bodegom PM, Aerts R, Callaghan TV, Van Logtestijn RSP, et al (2007) Global negative vegetation feedback to climate warming responses of leaf litter decomposition rates in cold biomes. Ecol Lett 10: 619–627. 17542940 10.1111/j.1461-0248.2007.01051.x

[14] Hansen AH, Jonasson S, Michelsen A, Julkunen-Tiitto R (2006) Long-term experimental warming, shading and nutrient addition affect the concentration of phenolic compounds in arctic-alpine deciduous and evergreen dwarf shrubs. Oecologia 147: 1–11. 16180043 10.1007/s00442-005-0233-y

[15] Sorensen PL, Michelsen A, Jonasson S (2008) Nitrogen uptake during one year in subarctic plant functional groups and in microbes after long-term warming and fertilization. Ecosystems 11: 1223–1233.

[16] Campioli M, Leblans N, Michelsen A (2012) Twenty-two years of warming, fertilisation and shading of subarctic heath shrubs promote secondary growth and plasticity but not primary growth. PLoS ONE 7: e34842 doi:10.1371/journal.pone.0034842. 22511968 10.1371/journal.pone.0034842 PMC3325270

[17] Flaig W (1964) Effects of micro-organisms in the transformation of lignin to humic substances. Geochim Cosmochim Ac 28: 1523–1535.

[18] Carreiro MM, Sinsabaugh RL, Repert DA, Parkhurst DF (2000) Microbial enzyme shifts explain litter decay responses to simulated nitrogen deposition. Ecology 81: 2359–2365.

[19] DeForest JL, Zak DR, Pregitzer KS, Burton AJ (2004) Atmospheric nitrate deposition, microbial community composition, and enzyme activity in northern hardwood forests. Soil Sci Soc Am J 68: 132–138.

[20] Edwards IP, Zak DR, Kellner H, Eisenlord SD, Pregitzer KS (2011) Simulated atmospheric N deposition alters fungal community composition and suppresses ligninolytic gene expression in a northern hardwood forest. PloS ONE 6: e20421 doi:10.1371/journal.pone.0020421. 21701691 10.1371/journal.pone.0020421 PMC3119081

[21] Havström M, Callaghan TV, Jonasson S (1993) Differential growth responses of Cassiope tetragona, an arctic dwarf-shrub, to environmental perturbations among three contrasting high- and sub-arctic sites. Oikos 66: 389–402.

[22] Michelsen A, Rinnan R, Jonasson S (2012) Two decades of experimental manipulations of heaths and forest understory in the Subarctic. Ambio 41: 218–230. 22864696 10.1007/s13280-012-0303-4 PMC3535062

[23] Wardle DA (2002) Communities and Ecosystems: Linking the Aboveground and Belowground Components. Monographs in Population Biology 34. Princeton University Press, Princeton, New Jersey.

[24] Bååth E (2003) The use of neutral lipid fatty acids to indicate the physiological conditions of soil fungi. Microbial Ecol 45: 373–383. 10.1007/s00248-003-2002-y 12704558

[25] Deslippe JR, Hartmann M, Mohn WW, Simard SW (2011) Long-term experimental manipulation of climate alters the ectomycorrhizal community of *Betula nana* in Arctic tundra. Global Change Biol 17: 1625–1636.

[26] Wixon DL, Balser TC (2013) Toward conceptual clarity: PLFA in warmed soils. Soil Biol Biochem 57: 769–774.

[27] Sorensen PL, Lett S, Michelsen A (2012) Moss-specific changes in nitrogen fixation following two decades of warming, shading, and fertilizer addition. Plant Ecol 213: 695–706.

[28] Sørensen L, Holmstrup M, Maraldo K, Christensen S, Christensen B (2006) Soil fauna communities and microbial respiration in high Arctic tundra soils at Zackenberg, Northeast Greenland. Polar Biol 29: 189–195.

[29] Schmidt IK, Jonasson S, Michelsen A (1999) Mineralization and microbial immobilization of N and P in arctic soils in relation to season, temperature and nutrient amendment. Appl Soil Ecol 11: 147–160.

[30] Jonasson S, Castro J, Michelsen A (2006) Interactions between plants, litter and microbes in cycling of nitrogen and phosphorus in the Arctic. Soil Biol Biochem 38: 526–532.

[31] Neff JC, Townsend AR, Gleixner G, Lehman SJ, Turnbull J, et al (2002) Variable effects of nitrogen additions on the stability and turnover of soil carbon. Nature 419: 915–917. 12410307 10.1038/nature01136

[32] Waldrop M, Zak D (2006) Response of oxidative enzyme activities to nitrogen deposition affects soil concentrations of dissolved organic carbon. Ecosystems 9: 921–933.

[33] Berg B, Matzner E (1997) Effect of N deposition on decomposition of plant litter and soil organic matter in forest systems. Environ Rev 5: 1–25.

[34] Rinnan R, Bååth E (2009) Differential utilization of carbon substrates by bacteria and fungi in tundra soil. Appl Environ Microbiol 75: 3611–3620. 19363072 10.1128/AEM.02865-08 PMC2687315

[35] Alexander M (1977) Introduction to soil microbiology. New York: John Wiley and Sons. 467 p.

[36] Jastrow JD, Amonette JE, Bailey VL (2007) Mechanisms controlling soil carbon turnover and their potential application for enhancing carbon sequestration. Climatic Change 80: 5–23.

[37] Strickland MS, Rousk J (2010) Considering fungal:bacterial dominance in soils – Methods, controls, and ecosystem implications. Soil Biol Biochem 42: 1385–1395.

[38] Kuzyakov Y (2010) Priming effects: Interactions between living and dead organic matter. Soil Biol Biochem 42: 1363–1371.

[39] Forsius M, Posch M, Aherne J, Reinds G, Christensen J, et al (2010) Assessing the impacts of long-range sulfur and nitrogen deposition on arctic and sub-arctic ecosystems. AMBIO 39: 136–147. 20653276 10.1007/s13280-010-0022-7 PMC3357685

[40] Michelsen A, Jonasson S, Sleep D, Havström M, Callaghan TV (1996) Shoot biomass, δ13C, nitrogen and chlorophyll responses of two arctic dwarf shrubs to in situ shading, nutrient application and warming simulating climatic change. Oecologia 105: 1–12. 28307116 10.1007/BF00328785

[41] Ruess L, Michelsen A, Schmidt IK, Jonasson S (1999) Simulated climate change affecting microorganisms, nematode density and biodiversity in subarctic soils. Plant Soil 212: 63–73.

[42] Frostegård Å, Tunlid A, Bååth E (1991) Microbial biomass measured as total lipid phosphate in soils of different organic content. J Microbiol Meth 14: 151–163.

[43] Olsson PA, van Aarle IM, Gavito ME, Bengtson P, Bengtsson G (2005) 13C incorporation into signature fatty acids as an assay for carbon allocation in arbuscular mycorrhiza. Appl Environ Microbiol 71: 2592–2599. 15870350 10.1128/AEM.71.5.2592-2599.2005 PMC1087529

[44] Boschker HTS (2004) Linking microbial community structure and functioning: stable isotope 13C labeling in combination with PLFA analysis. In: Kowalchuk GA, de Bruijn FJ, Head IM, Akkermans AD, van Elsas JD (2004) editors. Molecular Microbial Ecology Manual II. Dordrecht: Kluwer Academic Publishers. 1673–1688.

[45] Abraham W-R, Hesse C, Pelz O (1998) Ratios of carbon isotopes in microbial lipids as an indicator of substrate usage. Appl Environ Microbiol 64: 4202–4209. 9797266 10.1128/aem.64.11.4202-4209.1998 PMC106628

[46] Frostegård A, Bååth E (1996) The use of phospholipid fatty acid analysis to estimate bacterial and fungal biomass in soil. Biol Fert Soils 22: 59–65.

[47] Frostegård Å, Tunlid A, Bååth E (2011) Use and misuse of PLFA measurements in soils. Soil Biol Biochem 43: 1621–1625.

